# First Report on the Synergistic Interaction between Essential Oils against the Pinewood Nematode *Bursaphelenchus xylophilus*

**DOI:** 10.3390/plants12132438

**Published:** 2023-06-25

**Authors:** Jorge M. S. Faria, Tomás Cavaco, Diogo Gonçalves, Pedro Barbosa, Dora Martins Teixeira, Cristina Moiteiro, Maria L. Inácio

**Affiliations:** 1 INIAV, I.P., National Institute for Agrarian and Veterinary Research, I.P., Quinta do Marquês, 2780-159 Oeiras, Portugal; tomasfcavaco@gmail.com (T.C.); diomascarenhas@gmail.com (D.G.); lurdes.inacio@iniav.pt (M.L.I.); 2 MED, Mediterranean Institute for Agriculture, Environment and Development & CHANGE—Global Change and Sustainability Institute, Institute for Advanced Studies and Research, Évora University, Pólo da Mitra, Ap. 94, 7006-554 Évora, Portugal; pedronematology@gmail.com; 3 Instituto Superior de Agronomia (ISA), Universidade de Lisboa, 1349-107 Lisboa, Portugal; 4 Centro de Química Estrutural, Institute of Molecular Sciences, Departamento de Química e Bioquímica, Faculdade de Ciências, Universidade de Lisboa, Campo Grande, 1749-016 Lisboa, Portugal; cmmoiteiro@fc.ul.pt; 5 HERCULES Laboratory, Évora University, Largo Marquês de Marialva 8, 7000-809 Évora, Portugal; dmt@uevora.pt; 6 Science and Technology School, Évora University, Rua Romão Ramalho nº 59, 7000-671 Évora, Portugal; 7 GREEN-IT Bioresources for Sustainability, Instituto de Tecnologia Química e Biológica, Universidade Nova de Lisboa (ITQB NOVA), Av. da República, 2780-157 Oeiras, Portugal

**Keywords:** biopesticide, essential oils, *Cymbopogon citratus*, *Foeniculum vulgare*, *Mentha piperita*, nematicide, pest management, pine wilt disease, *Satureja montana*, synergism

## Abstract

Control of the pinewood nematode (PWN), the causal agent of pine wilt disease, can be achieved through the trunk injection of nematicides; however, many pesticides have been linked to environmental and human health concerns. Essential oils (EOs) are suitable alternatives due to their biodegradability and low toxicity to mammals. These complex mixtures of plant volatiles often display multiple biological activities and synergistic interactions between their compounds. The present work profiled the toxicity of eight EOs against the PWN in comparison to their 1:1 mixtures, to screen for successful synergistic interactions. Additionally, the main compounds of the most synergistic mixtures were characterized for their predicted environmental fate and toxicity to mammals in comparison to emamectin benzoate, a commercial nematicide used against PWN. The mixtures of *Cymbopogon citratus* with *Mentha piperita* and of *Foeniculum vulgare* with *Satureja montana* EOs showed the highest activities, with half-maximal effective concentrations (EC_50_) of 0.09 and 0.05 µL/mL, respectively. For these, complete PWN mortality was reached after only ca. 15 min or 2 h of direct contact, respectively. Their major compounds had a higher predicted affinity to air and water environmental compartments and are reported to have very low toxicity to mammals, with low acute oral and dermal toxicities. In comparison, emamectin benzoate showed lower nematicidal activity, a higher affinity to the soil and sediments environmental compartments and higher reported oral and dermal toxicity to mammals. Overall, uncovering synergistic activities in combinations of EOs from plants of different families may prove to be a source of biopesticides with optimized toxicity against PWNs.

## 1. Introduction

Currently, the greatest challenge to global agroforestry is an increasing product demand from the growing human population coupled with the restoration of natural and agroecosystems, as a means to counter the adverse effects of climate change [1]. The expansion of trade activities around the globe has fueled a rising number of invasion episodes by viruses, bacteria, fungi, nematodes and insect herbivores [2]. The pinewood nematode (PWN), *Bursaphelenchus xylophilus* (Steiner & Bührer 1934), Nickle 1970 is the causal agent of pine wilt disease (PWD) and has become the highest threat to European pine forests, after its first detection in Portugal in 1999 [3]. This forest pathogen is believed to be endemic to North America, where its colonization is non-damaging to native pine species. However, the introduction of PWNs to the susceptible pine forests of Japan at the beginning of the 20th century generated massive ecological, economic and cultural impacts [4]. Since then, this phytoparasite has spread to China (1982), Korea (1988) and ultimately to Europe, and it is now considered among the top 10 phytoparasitic nematodes with the greatest economic and scientific significance [5].

The most common pest management strategies used against PWNs are the immediate elimination of infected trees and wood, the treatment of wood used for transport activities and the populational control of its insect vectors of the genus *Monochamus*, responsible for the rapid spread of this phytoparasite. Chemical control is amply employed, either indirectly, through the use of insecticides for reducing insect-vector populations, or directly, by eliminating PWNs through the trunk injection of nematicides [6]. However, synthetic pesticides can damage communities of beneficial microorganisms, accumulate above the regulated levels in soil and food plants and become harmful to humans and animals [7,8].

The overuse of conventional agrochemicals has weakened the agroecosystems’ resilience to biotic and abiotic stress and led to increased degradation of global productive systems. European directives for the coming years emphasize a low pesticide-input approach coupled with the development of sustainable alternatives for integrated pest management [9]. Research on bioactive essential oils (EOs) has greatly contributed to the development of new biochemical biopesticides. An EO is the concentrated hydrophobic liquid commonly obtained via the hydrodistillation of a plant or plant part. EOs are largely comprised of secondary metabolites, being generally composed of mono- and sesquiterpenes, phenylpropanoids and also other groups of compounds [10]. Their use as biopesticides has gained much attention, with several EOs showing a promising activity, in some cases higher than conventional nematicides [11]. To date, a total of 417 EOs extracted from 217 plants of 46 families have been analyzed against the PWN [12]. More than half belong to the Apiaceae, Lamiaceae, Myrtaceae and Rutaceae families, known for their abundance of aromatic and medicinal plant species. Remarkably, in more than 30% of these bioassays, strong activity was obtained against PWNs. The EOs with the highest nematicidal activity are mainly composed of oxygen- or sulfur-containing compounds, which may suggest a correlation between nematotoxic activity and the presence of electronegative elements [12]. However, the biological activity of an EO is, generally, the result of its complex mixture of volatiles, often displaying synergistic interactions, when the biological activity of the EO is greater than the sum of its volatiles’ activities; antagonistic interactions, when some compounds negatively influence the activity of the bioactive compounds; and additive interactions, when the biological activity of the EO is merely the sum of the activity of its components [13]. Synergistic interactions between compounds are often the result of an increase in the permeability of the plasmatic membrane to the bioactive EO compound or the enhancement of binding to transmembrane proteins, although the exact mechanism of action is often quite difficult to determine [14].

Given that these interactions have only been seldom exploited for increased nematicidal activity against the PWN, the present work focuses on uncovering the synergistic or antagonistic relationships between eight EOs from different plants, by screening the nematotoxic activity of 1:1 mixtures of EOs against PWNs. Synergistic interactions can contribute to enhancing the nematicidal activity of biopesticidal formulations as sustainable alternatives to commonly used nematicides.

## 2. Results

### 2.1. Volatile Profiles of the Essential Oils

The chemical composition of the EOs, determined through gas chromatography coupled with mass spectrometry (GC-MS), revealed a prevalence of monoterpenes, with the exception of *Foeniculum vulgare* (Apiaceae) EO, mainly composed of the phenylpropanoid *trans*-anethole (75%) (Table 1). For the EO of *Eucalyptus globulus* (Myrtaceae), the oxygen-containing monoterpene 1,8-cineole was the main compound, with a relative amount of 75%. The aldehyde monoterpene stereoisomers geranial (35%) and neral (22%), the alcohol geraniol (18%) and the hydrocarbon *β*-myrcene (20%) dominated the EO of *Cymbopogon citratus* (Poaceae). The EO of *Origanum vulgare* (Lamiaceae) showed high relative amounts of the monoterpene alcohols *α*-terpineol (42%), linalool (17%) and phenol thymol (13%). The EO of *Mentha piperita* (Lamiaceae), peppermint, was rich in menthone (57%) and pulegone (13%), two monoterpene ketones. For *Rosmarinus officinalis* EO (Lamiaceae), monoterpene hydrocarbons were more abundant than oxygen-containing monoterpenes, showing high proportions of *β*-myrcene (31%), α-pinene (16%) and verbenone (11%), a ketone. The EO of the Lamiaceae *Salvia officinalis* had high relative amounts of the monoterpene ketones *α*-thujone (30%) and *β*-thujone (10%), the cyclic ether 1,8-cineole (27%) and the hydrocarbon *α*-pinene (11%). The EO of winter savory, *Satureja montana* (Lamiaceae), was rich in carvacrol (64%), a monoterpene phenol, and the monoterpene hydrocarbon *γ*-terpinene (18%).

### 2.2. Nematicidal Activity of EOs and EO Mixtures

The nematicidal activity of EOs and their 1:1 mixtures was compared to that of the commercial nematicide Pursue^®^, currently used in Europe in the pest management of PWN. Pursue was assayed at 1 mg of emamectin benzoate (its active compound) per mL of methanol or ultrapure water, and induced strong mortalities, namely, 89.6 ± 0.9 and 88.9 ± 0.4%, respectively. In comparison, the EO of *O. vulgare* showed a similar strong activity (88.8 ± 0.3%) while that of *E. globulus*, *M. piperita*, *R. officinalis* and *S. officinalis* were mostly inactive when tested solely (bold values in Table 2). The EOs of *C. citratus*, *F. vulgare* or *S. montana* induced complete mortality (100%) at 1 µL/mL and were tested at lower concentrations. For these EOs, the half-maximal effective concentration (EC_50_) values were determined to characterize their toxicological strength (Table 2).

*Satureja montana* EO had the highest activity (0.15 ± 0.00 µL/mL) followed by *C. citratus* EO (0.29 ± 0.01 µL/mL) and lastly the EO of *F. vulgare* (0.51 ± 0.10 µL/mL) (Table 2).

**Figure 1:**
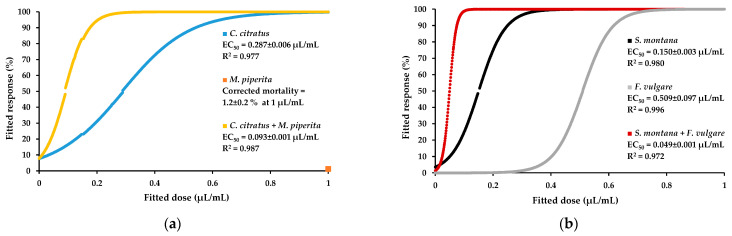
Graphical representation of the dose–response curves fitted for the corrected pinewood nematode mortality values obtained from decreasing concentrations of the most successful essential oil (EO) mixtures and respective component EOs. The EOs of *Mentha piperita* (orange), *Cymbopogon citratus* (blue) and its 1:1 combination (yellow) (**a**); and the EOs of *Satureja montana* (black), *Foeniculum vulgare* (grey) and its 1:1 combination (red) (**b**).

The 1:1 mixtures of EOs induced different toxicities on PWNs, with some showing additive interactions while others showed synergistic or antagonistic interactions (Table 2). For *C. citratus* EO, an antagonistic interaction was observed for its combination with the EO of *E. globulus*, not reaching complete activity at 1 µL/mL, while combinations with the remaining EOs induced EC_50_ values lower than that of their constituent EOs tested solely. The mixtures of *C. citratus* with *R. officinalis* (EC_50_ = 0.19 µL/mL) or *S. officinalis* (EC_50_ = 0.14 µL/mL) EOs resulted in an increase in activity; however, only its combination with *M. piperita* EO could be considered strongly synergistic (EC_50_ = 0.09 µL/mL), for inducing a ca. threefold lower EC_50_ value than that of *C. citratus* EO tested alone (Figure 1a). The combination of *E. globulus* EO with the other EOs was either additive or antagonistic, namely, its combination with the EOs of *C. citratus*, *F. vulgare* or *S. montana*. An antagonistic interaction was found between the EOs of *F. vulgare* and *R. officinalis*; however, synergistic interactions were also found, in its combination with *O. vulgare* (EC_50_ = 0.13 µL/mL) or *S. montana* (EC_50_ = 0.05 µL/mL), in which activity was 10-fold lower than the activity obtained for *F. vulgare* EO and 3-fold lower than the activity obtained for *S. montana* EO, tested solely (Figure 1b). Additionally, slightly synergistic interactions were found for the EOs of *M. piperita* with *O. vulgare*, and *O. vulgare* with the EOs of *R. officinalis* or *S. officinalis*.

For the most successful EO mixtures and respective constitutive EOs, the half-maximal effective time (ET_50_, in min) values along with the lowest maximal effective time (ET_100_, in min) and lowest maximal effective concentration (EC_100_, in µL/mL) values were determined (Table 3). The fastest-acting EO was that of *S. montana* with an ET_50_ of 2.5 min, followed by the mixture of *C. citratus* with *M. piperita* EOs, the EO of *C. citratus* applied solely, the mixture of *S. montana* with *F. vulgare* EOs and lastly by the EO of *F. vulgare* applied alone (Table 3). The lowest direct contact time required to fully immobilize the PWN population (ET_100_) was obtained for *S. montana* EO, followed by the combination of *C. citratus* with *M. piperita* EOs, the EO of *C. citratus*, the combination of *S. montana* and *F. vulgare* EOs and, finally, *F. vulgare* EO tested alone. The lowest concentration required to eliminate 100% of the PWN population was found for the combination of *S. montana* and *F. vulgare* EOs, followed by *S. montana* EO, the combination of *C. citratus* with *M. piperita* EOs, *F. vulgare* EO and lastly the EO of *C. citratus* (Table 3).

### 2.3. Potential Environmental Fate and Human Health Impacts of the Main EO Volatiles

To estimate the environmental fate of the most active EO mixtures (EOs of *S. montana* with *F. vulgare* and EOs of *C. citratus* with *M. piperita*), the predicted environmental distribution (PED) of their main EO compounds (≥5%), namely, *trans*-anethole, carvacrol, geranial, geraniol, menthone, *β*-myrcene, neral, pulegone, *α*-pinene and *γ*-terpinene, was compared to that of emamectin benzoate (Table 4). Information on the isomers geranial and neral was mainly found for their mixture, designated as citral, which is more commonly found in natural conditions. The predicted environmental fate of each EO compound differed greatly from that of the (hemi)synthetic emamectin benzoate. Due to their volatile nature, EO compounds were predicted to be favorably distributed in the air environmental compartment, with percentages that varied from 19 (citral) to 100% (*α*-pinene and *β*-myrcene), with the exception of carvacrol (2%). On the other hand, emamectin benzoate showed a very low predicted affinity to this compartment (ca. 50 parts per million) (Table 4). For the water environmental compartment, the greatest affinities were predicted for citral (68%), geraniol (65%), *trans*-anethole (28%), pulegone (27%), carvacrol (23%) and menthone (22%), while for the remaining compounds, the percentages were below 0.5%. The predicted distribution of emamectin benzoate in the soil environmental compartment was the highest of all the analyzed compounds (ca. 98%), followed by carvacrol (73%), pulegone (39%), *trans*-anethole (30%), geraniol (13%) and citral (12%), with the remaining compounds showing percentages below 6% (Table 4). In the environmental compartment of the sediments, the EO volatiles obtained a consistently lower percentage (between 0.003%, for *β*-myrcene, and 1.6%, for carvacrol) than emamectin benzoate (2.17%).

To understand the potential safety for human health in the usage of EO mixtures, the oral and dermal toxicity thresholds for mammals were retrieved from online databases and compared to those of emamectin benzoate. The values of lethal doses, LD_50_, mg/kg, were shown to be consistently higher for EO compounds than for the commercial nematicide (Table 4), indicating their lower toxicity for mammals. For acute oral toxicity, LD_50_ values varied from 7-, for pulegone, to 108-fold, for citral, higher than the value reported for emamectin benzoate. For acute dermal toxicity, the LD_50_ values ranged from >1000 mg/kg, for citral, to >5000 mg/kg, for *α*-pinene, *trans*-anethole, geraniol or *β*-myrcene, which is roughly 2- to 11-fold the LD_50_ value reported for emamectin benzoate (439 mg/kg) (Table 4).

## 3. Discussion

The tested EOs showed quantitative and qualitative differences in their volatile compositions, which influenced the nematicidal activity of each EO. The most active EOs, namely, those of *C. citratus*, *F. vulgare* and *S. montana,* were rich in oxygen-containing compounds, namely, *trans*-anethole, carvacrol, geranial, geraniol and neral; however, compounds without oxygen were also present in high amounts, namely, *β*-myrcene and *α*-pinene. This suggests that nematicidal activity was imposed by specific oxygen-containing compounds, i.e., compounds with electronegative elements. This same effect has been reported in a previous work, where the oxygen-containing molecules from EOs with high activity against PWNs were separated from the respective hydrocarbon molecules [18]. In fact, this characteristic appears to be transversal to the majority of reports on the use of EOs against PWNs [12]. Nevertheless, in the present work, the EOs with the lowest activities against PWNs, namely, those of *E. globulus*, *M. piperita*, *R. officinalis* and *S. officinalis*, also had high amounts of oxygen-containing molecules in their compositions, e.g., 1,8-cineole, menthone, pulegone, *α*-thujone, *β*-thujone and verbenone, and, again, the monoterpene hydrocarbons *β*-myrcene and *α*-pinene were present in high amounts. In fact, the major oxygen-containing compounds of the EOs with the highest activity had phenol, aldehyde and alcohol functional groups, while the oxygen-containing compounds of the EOs with the lowest activities were cyclic ether and ketones, suggesting that volatiles from the former groups may induce higher activities than the latter against PWNs. A similar observation was previously reported for the screening of commercially acquired monoterpenes against PWNs [19]. In this work [19], the phenols carvacrol and thymol (positional isomers), the alcohols geraniol, nerol (*cis* and *trans* isomers), menthol and citronellol and the aldehydes citronellal and citral (geranial and neral) showed higher activities than the tested hydrocarbons, e.g., *β*-myrcene, *α*-pinene or *β*-pinene, or ketones, e.g., menthone, pulegone or carvone. Additionally, the trunk injection nematicide levamisole hydrochloride was tested as a positive control, showing lower activity than the most active monoterpenes. This nematicide has a high affinity to soil and sediment environmental compartments and is reported to have a low LD_50_ value for oral toxicity (180 mg/kg), similarly to emamectin benzoate [15,16,17,20].

The EOs of *C. citratus* have been previously tested against PWNs with good results [11,18,21,22]. In these works, the EO volatile profiling, when performed, showed a composition in major volatiles like the one of the EO used in the present study. The nematicidal activity was very similar to that reported in the present study at the tested concentrations; however, the EC_50_ values, when determined, were slightly higher, suggesting that small variations in compound amounts may cause variability in the activity against PWNs. Additionally, a visual assessment of nematode mortality under a microscope can be easily prone to variability being heavily dependent on the observers and their experience.

For *F. vulgare*, the EOs tested in previous works showed volatile compositions similar to that of the one used in the present work but induced only low-to-moderate mortalities in PWNs, at 2 µL/mL [18,22,23]. The EO that showed the highest activity (a 66% corrected mortality at 2 µL/mL) was tested with methanol as a solubilizer, similarly to the present work, which might suggest an influence of the solubilizer compound on the activity of some EOs in direct-contact bioassays. In fact, a previous work compared the suitability of two solubilizer compounds with different chemical characteristics, Triton X-100, a nonionic detergent-type surfactant, and acetone, a polar aprotic organic solvent, and found that, for some EOs, the solubilizer strongly influenced their activity against PWNs, while in others, only slight differences were reported [23]. This indicates that the combination of EO compounds with a solubilizing agent can have a strong influence on the observed nematicidal activity of the EO.

The EOs of *S. montana* assayed in previous works showed quantitative variations in their volatile composition which appeared to influence the activity against the PWN [18,22,23]. In bioassays at 2 and 1 µL/mL, *S. montana* EOs showed complete mortality (100%); however, at 0.5 µL/mL, while one EO (with 64% of carvacrol and 18% of γ-terpinene) showed 60% mortality; the other EO (with 40% of carvacrol, 20% of *p*-cymene and 15% of thymol, a positional isomer of carvacrol) showed only 25% corrected mortality, indicating that the amounts of carvacrol, γ-terpinene and/or *p*-cymene can influence activity against PWNs.

Similarly to the present study, the anti-PWN activities of *O. vulgare* EOs reported in previous works varied from strong (80%) to complete (100%), in bioassays using 1, 2 or 10 µL (or mg)/mL [11,18,22]. The chemical profiling of the reported EOs showed varying amounts of *α*-terpineol (40%), carvacrol (10–36%), linalool (16%) and thymol (12–15%).

Concerning the EOs with low activities, in previous reports, *E. globulus* EO showed mortalities that varied from 0 to 4%, at 2 to 10 µL (or mg)/mL, being mainly rich in 1,8-cineole and *α*-pinene; *M. piperita* EO showed mortalities that varied from 46 to 85%, at 2 µL/mL, with high amounts of menthone, menthol and pulegone; *R. officinalis* EO showed mortalities that varied from 0 to 31%, at 2 to 10 µL (or mg)/mL, being rich in *β*-myrcene and α-pinene; while *S. officinalis* EOs showed mortalities that varied from 0 to 16%, at 2 to 10 µL (or mg)/mL, being mainly composed of α-thujone, 1,8-cineole and *β*-thujone [11,18,21,22,23].

The synergistic interactions in mixtures of EOs were determined for the first time for the PWN. Additive, synergistic, and antagonistic interactions were detected in varying degrees. Combinations featuring the EO of *E. globulus* showed either additive or antagonistic interactions, indicating that its volatiles, mainly 1,8-cineole and *α*-pinene, may have some inhibitory activity on other nematicidal EO compounds, against PWNs. In fact, in a study on the interactions of EO compounds with activity against the yellow fever mosquito *Aedes aegypti*, responsible for the transmission of serious human diseases, 1,8-cineole (eucalyptol) showed mainly antagonistic interactions with other monoterpenes, e.g., carvone and limonene, but synergistic interactions with *α*-pinene, while *α*-pinene showed either antagonistic or no interaction with other terpenes [24]. However, in a study on the interactions of several characteristic EO compounds against *Spodoptera littoralis* larvae, most synergistic interactions were attributed to six compounds, among them, *trans*-anethole, *γ*-terpinene and *p*-cymene [25]. Most notably, synergistic interactions were found for binary mixtures of *trans*-anethole with carvacrol or γ-terpinene, α-pinene with γ-terpinene and *β*-myrcene with menthone. In a follow-up study on these EO compounds against *Culex quinquefasciatus* larvae, strong synergistic interactions were found for the binary mixtures of *β*-myrcene with menthone and *trans*-anethole with γ-terpinene, the binary mixtures of carvacrol with *trans*-anethole or α-pinene showed milder synergistic interactions [26].

In the present work, the strongest synergistic interactions were obtained for the combination of *S. montana* with *F. vulgare* EOs, suggesting the existence of this interaction between the main components of each EO, namely, *trans*-anethole with carvacrol and/or *γ*-terpinene. In a study analyzing the interactions of EO compounds against the animal parasitic nematode *Haemonchus contortus*, 1:1 mixtures of carvacrol with cinnamaldehyde, a phenylpropanoid, or thymol, its isomer, showed synergistic interactions; its 1:1 combination with carvone (as well as the combination thymol/carvone) showed an antagonistic interaction; however, its interaction with *trans*-anethole was only additive [27]. On the other hand, *trans*-anethole showed a synergistic interaction with carvone but additive with thymol and cinnamaldehyde. In another study, where mixtures of EO compounds were bioassayed against the phytoparasitic nematode *Meloidogyne incognita*, *F. vulgare* EO’s main compound *trans*-anethole showed synergistic interactions with carvacrol (at 2:1.25 ratio) or its isomer thymol (at 3:1.25 ratio) [13], suggesting a specificity on synergistic interactions with regard to the nematode group.

Another highly synergistic combination detected in the present study was that of *C. citratus* with *M. piperita* EOs. Synergistic interactions have been reported before for *C. citratus* EO main compounds geranial and neral [28]. In this study, citral was profiled in *C. citratus* EO with relative amounts of 43% for geranial and 33% for neral. Additionally, its combination with 2-undecanone, an aliphatic ketone commonly present in the EO of *Ruta graveolens*, in a 2:1 ratio resulted in a strong synergistic activity against the phytoparasitic nematode *M. incognita*, in bioassays in vitro, through direct-contact bioassays, or in vivo, using potted tomato plants in controlled greenhouse conditions. In another study that screened the interaction of EO compounds at different ratios, pulegone, a ketone characteristic of *M. piperita* EO, in a 1:2 combination with geraniol, commonly found in *C. citratus* EO, was reported to show only an additive effect, which may indicate a specificity in the ratios of compounds with regard to their nematicidal interactions [13].

In the present study, the time required for complete mortality was determined for the first time in the bioassay of EOs and EO mixtures against PWNs. This parameter is very important for the development of bionematicides since a compromise must be found between the time that the EO takes to exert its effect and its characteristic volatility or biodegradability. Surprisingly, the most successful EOs and their mixtures appear to act quite quickly, enforcing their potential for the development of successful biopesticides. In other studies, the shortest bioassay duration was 4 h, with the EOs of *Allium sativum* (composed of diallyl sulfide, diallyl disulfide and diallyl trisulfide) showing complete mortality at 0.0625 mg/mL and *Allium cepa* (composed of propyl trisulfide, propyl disulfide, methyl propyltrisulfide and methyl propyldisulide) showing EC_50_ values as low as 0.0121 mg/mL [21,29]. However, lower bioassay time periods were not bioassayed.

Despite the successful activities shown by EO mixtures, their potential use for the innovation of bionematicides also depends on higher safety for the environment and low toxicity to humans, in comparison to the currently used nematicides. Generally, the environmental risk assessment of pesticide compounds mainly depends on combining data on exposure and effects. In the case of exposure to the environment, the Mackay fugacity models are a good approach to predict the affinity of a stressor to the several environmental compartments, using established in silico computations, while for the data on the effects, several online databases offer compilations of data on experimental assays, conducted in standard conditions, that evaluate acute or chronic toxicities. In the present work, as a contribution to this evaluation, the predicted environmental distribution of the main EO compounds as well as their reported acute oral and dermal toxicities were compared to those of the commercial nematicide’s active substance. The EO volatiles were mainly predicted to be dispersed to either the atmosphere or water deposits; furthermore, their toxicity to mammals was consistently lower than that of emamectin benzoate. This nematicide has a high affinity with soil and sediments and also higher toxicity to mammals than EO volatiles. In fact, the analyzed EO compounds are currently categorized as flavoring agents, additives that improve aroma or taste and have been approved for safe human consumption [30]. For example, for *C. citratus* EOs tested on rats, with daily intake over 14 days, only doses above values as high as 1500 mg/kg were seen to exert any functional damage to the stomach and liver [31]. Additionally, being authorized for human consumption is known to greatly facilitate the process of approval for new bionematicides or plant protection products (PPPs), when compared with synthetic compounds [32].

Concerning their environmental safety, although a great affinity was predicted for the air and water environmental compartments, the EO compounds are volatile and highly biodegradable, which leads to very low recalcitrance in the environment. Additionally, citral and geraniol have a higher affinity to the water environmental compartment; however, their reported LC_50_ for fish is about 6.1 mg/L and 11.6 mg/L, respectively, a higher value than 0.174 mg/L, reported for emamectin benzoate [16]. The same tendency is reported for other aquatic test model organisms, which shows that the compounds with a higher risk of spreading to the aquatic biota, due to their higher affinity for this compartment, are reportedly less toxic than emamectin benzoate. Concerning the air environmental compartment, ecotoxicological information was found for pollinator insects, for example, the honeybee (*Apis mellifera*). While emamectin benzoate has a reported LD_50_ of 0.036 μg/bee, the monoterpenes carvone or citral have extremely higher values, 106,620 μg/bee and 78,459 μg/bee, respectively, which even makes them potential biopesticides able to control *Varroa destructor*, a highly damaging ectoparasite of honeybees [16,33]. Ultimately, the identified EO mixtures can offer a safer alternative to conventional nematicides while showing improved nematicidal activity against PWNs.

## 4. Materials and Methods

### 4.1. Essential Oils

Direct-contact bioassays were performed with the EOs of eucalypt leaves (*Eucalyptus globulus*), fennel shoots (*Foeniculum vulgare*), lemongrass leaves (*Cymbopogon citratus*), oregano flowering shoots (*Origanum vulgare*), peppermint shoots (*Mentha piperita*), rosemary flowering shoots (*Rosmarinus officinalis*), sage shoots (*Salvia officinalis*) and winter savory flowering shoots (*Satureja montana*), acquired from certified local retail sellers.

### 4.2. Chemical Profiling of EOs and EO Mixtures

The chemical composition of EOs and their mixtures was profiled with a Shimadzu GC2010 gas chromatographer coupled to a GC-MS-QP2010 Plus mass spectrometer (Shimadzu, Kyoto, Japan), by injecting 0.1 µL of a sample of each EO diluted (1:1, *v*/*v*) in *n*-hexane (95%, Optima grade for HPLC and GC-MS, Fisher Chemicals, Hampton, NH, USA). The chromatographic separation was performed with a Zebron column ZB-5HT (30 m length, 0.25 mm I.D., 0.25 μm film thickness) (Phenomenex, Torrance, CA, USA). Injections were performed using a split sampling technique (ratio 1:100) with the injector temperature set to 250 °C and a helium (He) flow of 1.5 mL/min. The GC oven temperature program was set to increase from 45 to 175 °C, at 3 °C/min, and then up to 300 °C, at 15 °C/min, with a final isothermal step for 10 min [18]. The mass spectrometer was operated in EI mode (70 eV) and scanned from 40 to 850 m/z. The ion source temperature was set at 240 °C, and the interface temperature was maintained at 280 °C. Peak assignment was performed using the National Institute of Standards and Technology (NIST), Wiley and laboratory-built mass spectra libraries, through AMDIS software (National Institute of Standards and Technology of the US Department of Commerce, Gaithersburg, MD, USA).

### 4.3. In Vitro Culturing of Pinewood Nematodes

To obtain large quantities of pinewood nematodes for the direct-contact bioassays, the isolate Bx0.13.003 was used, which is a reference isolate kept at the Plant Nematology Lab of the National Institute for Agrarian and Veterinary Research (INIAV, I.P.) at Oeiras, Portugal, for research purposes. Bx0.13.003 was isolated from a *Pinus pinaster* field tree displaying strong PWD symptomatology (N 39°43′338″, W 9°01′557″). An internal transcribed spacer (ITS) region sequence was deposited in the GenBank database (NCBI) with the accession number MF611984.1. Larger quantities of this PWN isolate were obtained by culturing in a non-sporulating *Botrytis cinerea* (de Bary) Whetzel strain in aseptic conditions. For this purpose, axenic cultures of *B. cinerea* were established on steam-sterilized hydrated certified organic commercial barley grains (*Hordeum vulgare* L.) (ca. 15 g cereal/15 mL ultrapure water, in 250 mL Erlenmeyer flasks) for 7 to 10 days at 25 ± 1 °C. Fungal mats, obtained after the surface of the cereal was fully colonized, were inoculated with 1 mL of a mixed-life-stage PWN suspension (1000 PWNs/mL) and kept at 25 ± 1 °C in darkness for 7 to 10 days until the fungal mat was consumed. To prevent unwanted microbial contamination that might influence mortality, the nematodes were surface sterilized before the last subculturing with an ethanol solution in ultrapure water (50% *v*/*v*) for 5 min [34] and then re-established on axenic mycelial mats. Nematodes were extracted using the modified Baermann funnel technique [35]. Aqueous solutions of PWNs were used for the direct contact assays, for further inoculations, or stored at 11 °C. The assessment of PWN numbers and/or survival rates was performed using an Olympus SZX12 (Tokyo, Japan) stereomicroscope (40×).

### 4.4. Nematicidal Activity of the Essential Oils and EO Mixtures

Direct-contact bioassays were performed in flat-bottom 96-well microtiter plates (Carl Roth GmbH & Co. KG, Karlsruhe, Germany). In each well, 95 µL of an aqueous suspension of mixed-life-stage PWNs (80–100 PWNs) were added to 5 µL of EO stock solution (prepared in HPLC-grade methanol, Fisher Chemicals, Hampton, NH, USA, at 20 µL/mL), to obtain a final EO concentration of 1 µL/mL. Blank wells were added with 5 µL of ultrapure water instead of the EO stock solution, to assess natural PWN mortality, and control wells with 5 µL of methanol, to determine mortality caused by the organic solvent. The microtiter plates were sealed with plastic film to prevent EO volatilization and mixed in an orbital shaker (IKA labortechnik, Staufen, Germany) at 800 cycles/min for 1 min. Plates were covered with aluminum foil to establish complete darkness and maintained for 24 h at 50 r.p.m. in an orbital shaker at 25 ± 1 °C. To determine EO nematicidal strength, live and dead PWNs were counted under a stereomicroscope (40×). To ascertain mortality, physical prodding was used on motionless PWNs to stimulate movement. If no movement was detected PWNs were considered dead. Three separate trials were performed for each sample in a total of 10 bioassays. EOs that showed full mortality were screened at lower EO concentrations (0.5, 0.25, 0.13, 0.06 and 0.03 µL/mL obtained by serial dilutions with a dilution factor of two) to determine toxicity thresholds.

For EO mixtures, stock solutions of combined EOs (2 per mixture) were prepared in methanol as described above. For each combination’s stock solution, individual EOs were added for a final concentration of 20 µL/mL each (20 µL/mL of EO #1 and 20 µL/mL of EO #2). Lower EO combination concentrations were obtained by serial dilutions with a dilution factor of two.

To determine the effective time thresholds for activity, the mortality of the most successful EOs and EO mixtures was determined at ca. 5, 30, 240 and 1440 min after application, at the lowest tested concentration that caused complete mortality (100%).

### 4.5. Predicted Environmental Fate of the Main Volatiles of the Most Successful EO Mixtures

The potential environmental fate of the main EO compounds identified in the EO mixtures with the highest nematicidal activity was determined through the predictive equilibrium criterion model suggested by Mackay et al. [15] and compared to that of the synthetic nematicide emamectin benzoate. Supported by this model, predicted environmental distribution (PED) percentages were obtained for each compound in the environmental compartments of air, water, soil and sediments by using the freely available Level I Mackay Fugacity Model beta version 4.31, Trent University, Canada [20]. The model for this level predicts a situation in which a fixed quantity of compound, namely, an illustrative 100,000 kg, is introduced in a closed system, under steady-state and equilibrium conditions, at a temperature of 25 °C. The chemical parameters needed from each compound, namely, molecular mass (g/mol), melting point (°C), vapor pressure (Pa), solubility in water (mg/L), air–water partition coefficient or Henry’s Law constant (Pa.m^3^/mol), n-octanol/water partition coefficient (log value of Kow) and soil organic carbon/water partition coefficient (Koc) were retrieved from the PubChem online database [16] and the PPDB: the Pesticide Properties Database [17] (Table 5).

### 4.6. Toxicity to Mammals of the Main Volatiles of the Most Successful EO Mixtures

Data on the toxicological parameters of the main EO compounds identified in the highest nematicidal EO mixtures and the synthetic nematicide emamectin benzoate on mammals were retrieved from PubChem [16] and PPDB: the Pesticide Properties Database [17].

### 4.7. Data Treatment and Statistical Analysis

Nematode mortality percentages were determined according to the formula: mortality% = [(dead PWNs)/(live + dead PWNs)] × 100. For each EO or EO combination concentration, corrected mortality percentages were determined using the formula: corrected mortality% = [(mortality% in treatment − mortality% in control)/(100 − mortality% in control)] × 100. The categorization established by Kong et al. [11] was used to classify the toxicological strength at each concentration, by considering mortality as complete when at 100%, strong when above 80%, moderate between 80 and 61%, weak between 60 and 40%, and low or inactive below 40%.

The determination of the half-maximal effective concentration (EC_50_) values was performed with Version 2019 of Origin Graphing and Analysis software (OriginLab, Northampton, MA, USA). A nonlinear regression analysis was performed by plotting corrected mortality values along EO or EO combination concentration values, and fitting a dose–response log-logistic equation: y = C + (D − C)/1 + exp {b [log (x) − log (EC_50_)]} [36], where C and D are the lower and upper limits of the sigmoidal dose–response curve, respectively; b is the slope, and EC_50_ is the EO concentration which induces a response halfway between the lower and upper limits. The upper (D) and lower (C) limits were set to 0 and 100%, respectively. For the assessment of synergistic and antagonistic interactions, the activities of the EO mixtures were compared to the sum of single EO activities, as described by Pavela [26] and Faraone et al. [37]. For the determination of the half-maximal effective time (ET_50_) values, the analysis parameters described above were equally applied except for the time values being plotted against the corrected mortality values, at the highest EO or EO mixture concentrations that induced complete mortality (100%). Thus, ET_50_ is the time that induces a response halfway between the lower and upper limits. The determination of the lowest maximal effective concentration (EC_100_) and lowest maximal effective time (ET_100_) was performed by solving the curve equation to the first y value of 100% mortality.

## 5. Conclusions

Essential oils are known to show strong nematicidal activities. Some are even used against plant parasitic nematodes, either whole or using their main compounds, in commercialized nematicidal formulations. Improving their nematicidal activity by combining EOs from plants of different families was tested for the first time for the PWN. When compared to the currently commercialized nematicides, the EO mixtures of *Cymbopogon citratus* with *Mentha piperita* and of *Foeniculum vulgare* with *Satureja montana* were strongly and quickly active, and they were predicted to volatilize to the atmosphere and have low toxicity for mammals, which turns them into strong candidates for the development of environmentally friendly biopesticides.

## Figures and Tables

**Table 1:** Main composition (compounds ≥ 1%, in relative amounts) of the essential oils (EOs) of eucalypt (*Eucalyptus globulus*), fennel (*Foeniculum vulgare*), lemongrass (*Cymbopogon citratus*), oregano (*Origanum vulgare*), peppermint (*Mentha piperita*), rosemary (*Rosmarinus officinalis*), sage (*Salvia officinalis*) and winter savory (*Satureja montana*), determined through gas chromatography–mass spectrometry (GC-MS).

EO Compounds * (≥1%)	*Cymbopogon citratus*	*Eucalyptus globulus*	*Foeniculum vulgare*	*Mentha piperita*	*Origanum vulgare*	*Rosmarinus officinalis*	*Salvia* *officinalis*	*Satureja montana*
*trans*-Anethole			75					
*β*-Bisabolene					2			
1,8-Cineole		75		3		4	27	
Camphene						4	3	
Camphor						8	6	
Carvacrol					6			64
*p*-Cymene					4	3		8
*β*-Caryophyllene	1			1	4	2	3	2
Geranial	35							
Geraniol	18							
Isomenthone				4				
Limonene		3		2		7		
Linalool					17	1		
Menthofuran				9				
Menthol				6				
Menthone				57				
*β*-Myrcene	20		1			31	2	1
Neomenthol				2				
Neral	22							
Pinocarvone		2						
*trans*-Pinocarveol		3						
Pulegone				13				
*α*-Phellandrene			8			2		
*α*-Pinene		16	13			16	11	1
*β*-Phellandrene						4		
*β*-Pinene			1	1			5	
Terpinen-4-ol					2	1		
Thymol					13			
*α*-Terpinene								2
*α*-Terpineol					42	3		
*α*-Thujone							30	
*β*-Thujone							10	
*γ*-Terpinene					8	2		18
Verbenone						11		
Viridiflorol							2	
Monoterpene hydrocarbons	20	19	24	3	12	68	20	31
Oxygen-containing monoterpenes	76	80		94	80	28	73	64
Sesquiterpene hydrocarbons	1			1	6	2	3	2
Oxygen-containing sesquiterpenes							2	
Phenylpropanoids			75					

* Values in the table are relative amounts of compounds in percentage (%); identification was based on National Institute of Standards and Technology (NIST), Wiley and laboratory-constructed mass spectra libraries; for compound Kovats indices, see Supplementary Material, Table S1.

**Table 2:** Nematicidal activity of the essential oils (EOs), on diagonal, and EO mixtures, below diagonal, expressed through their half-maximal effective concentration (EC_50_, in µL/mL) or corrected mortality (%) values at 1 µL/mL.

EOs	*Cymbopogon citratus*	*Eucalyptus globulus*	*Foeniculum vulgare*	*Mentha piperita*	*Origanum vulgare*	*Rosmarinus officinalis*	*Salvia* *officinalis*	*Satureja montana*
*C. citratus*	0.287 ± 0.006 ^1^							
*E. globulus*	**97.4 ± 0.9 ^2^**	**6.7 ± 0.2**						
*F. vulgare*	0.200 ± 0.002	**77.9 ± 1.0**	0.509 ± 0.097					
*M. piperita*	0.093 ± 0.001	**18.7 ± 1.0**	**94.7 ± 1.8**	**1.2 ± 0.2**				
*O. vulgare*	0.205 ± 0.005	**87.5 ± 1.9**	0.131 ± 0.004	0.194 ± 0.006	**88.8 ± 0.3**			
*R. officinalis*	0.185 ± 0.002	**13.0 ± 0.9**	**64.3 ± 1.7**	**51.8 ± 2.8**	0.250 ± 0.005	**14.2 ± 0.9**		
*S. officinalis*	0.139 ± 0.002	**8.3 ± 0.6**	**96.8 ± 0.7**	**12.6 ± 0.8**	0.141 ± 0.002	**12.9 ± 1.0**	**3.0 ± 0.2**	
*S. montana*	0.161 ± 0.001	0.186 ± 0.003	0.049 ± 0.001	0.139 ± 0.002	0.137 ± 0.005	0.136 ± 0.005	0.142 ± 0.004	0.150 ± 0.003

^1^ For 95% confidence intervals, see Supplementary Material, Table S2; ^2^ values in bold are for the corrected mortality (%) values of EOs that did not induce complete mortality at 1 µL/mL.

**Table 3:** Half-maximal effective time (ET_50_, in min, average ± standard error), lowest maximal effective time (ET_100_, in min; with 95% confidence intervals) and lowest maximal effective concentration (EC_100_, in µL/mL; with 95% confidence intervals) of the most successful essential oils (EO) or EO mixtures.

EOs/EO Mixtures	ET_50_ (min) (Average ± Standard Error)	ET_100_ (min) (95% Confidence Interval)	EC_100_ (µL/mL) (95% Confidence Interval)
*Cymbopogon citratus*	11.244 ± 6.005 ^1^	28.111 ^1^ (7.207–33.153)	0.817 (0.762–0.861)
*C. citratus* + *M. piperita*	5.851 ± 3.561 ^1^	14.628 ^1^ (4.324–17.297)	0.614 (0.553–0.639)
*Foeniculum vulgare*	73.918 ± 2.399 ^1^	747.057 ^1^ (559.279–789.910)	0.701 (0.285–0.963)
*Satureja montana*	2.582 ± 0.478 ^2^	6.454 ^2^ (2.883–7.207)	0.464 (0.413–0.491)
*S. montana* + *F. vulgare*	14.828 ± 0.366 ^3^	123.060 ^3^ (92.252–129.730)	0.208 (0.144–0.219)

^1^ Determined at 1 µL/mL, ^2^ determined at 0.5 µL/mL, ^3^ determined at 0.25 µL/mL.

**Table 4:** Main compounds of the nematicidal essential oil mixtures (≥5%) determined through gas chromatography–mass spectrometry (GC-MS), their predicted environmental distribution (PED) percentages in the air, water, soil and sediments environmental compartments computed through the Mackay fugacity model [15], and their oral and dermal acute toxicities for mammals (median lethal dose, LD_50_, mg/kg) obtained from PubChem online database [16] and PPDB: the Pesticide Properties Database [17].

EO Mixtures (1:1)	Compounds (%, in Relative Amounts)	Predicted Environmental Distribution (PED, %)				Toxicity to Mammals (LD_50_, mg/kg)	
		Air	Water	Soil	Sediments	Oral	Dermal
*S. montana* + *F. vulgare*	carvacrol (39)	1.7	23.2	73.4	1.6	810	2700
	*trans*-anethole (36)	41.1	27.9	30.2	0.7	3050	>5000
	*γ*-terpinene (7)	96.4	0.2	3.3	0.1	3650	>2000
	*α*-pinene (5)	99.7	0.5	0.3	0.0	3700	>5000
*C. citratus* + *M. piperita*	citral (27, geranial + neral)	19.1	68.4	12.3	0.3	6800	>1000
	menthone (23)	72.1	22.1	5.7	0.1	500	2180
	geraniol (10)	21.8	65.3	12.7	0.3	>4000	>5000
	*β*-myrcene (10)	99.8	0.1	0.1	0.0	>3380	5000
	pulegone (9)	32.6	27.2	39.3	0.9	470	3090
Pursue*^®^* ^1^	emamectin benzoate	5.0 × 10^−6^	0.1	97.6	2.2	63	439

^1^ For comparison purposes the commercial nematicide Pursue^®^ was used, in which emamectin benzoate is the active compound.

**Table 5:** Physical and chemical properties of the main EO compounds identified for most nematicidal EO mixtures, required to perform the Level I Mackay Fugacity Model [20] [molecular mass (g/mol), melting point (°C), vapor pressure (Pa), solubility in water (mg/L), air–water partition coefficient or Henry’s Law constant (Pa.m^3^/mol), n-octanol/water partition coefficient (logKow) and soil organic carbon/water partition coefficient (Koc). Data were retrieved from the PubChem database [16] and PPDB: the Pesticide Properties Database [17].

Main EO Compounds	CAS Number	Molecular Mass (g/mol)	Melting Point (°C)	Vapor Pressure (Pa)	Solubility in H_2_O (mg/L)	Henry’s Law Constant (Pa.m^3^/mol)	logK_OW_ (Unitless)	K_OC_ (Unitless)
*α*-pinene	80-56-8	136.23	−63	633.281	2.49	10,841.775	4.83	2600
*trans*-anethole	4180-23-8	148.20	21	6.666	111.00	7.275	3.30	500
carvacrol	499-75-2	150.22	1	3.090	1250.00	0.371	3.33	1469
*γ*-terpinene	99-85-4	136.23	−10	0.145	8.68	2280.770	4.50	8035
citral ^1^ (geranial + neral)	5392-40-5	152.23	−10	12.172	1340.00	1.383	2.76	83
geraniol	106-24-1	154.25	−15	4.000	100.00	1.652	2.90	90
menthone	14073-97-3	154.25	−6	35.997	688.00	16.212	3.05	120
*β*-myrcene	123-35-3	136.23	−80	267.978	4.09	9281.370	4.33	1074
pulegone	89-82-7	152.23	25	12.399	276.00	5.948	3.08	670
emamectin benzoate	155569-91-8	1008.2	144	5 × 10^−6^	24.00	0.0002	5.00	377,500

^1^ Citral occurs as a mixture of the two geometric stereoisomers geranial (*trans*-citral) and neral (*cis*-citral), more commonly found in natural conditions.

## Data Availability

The raw data supporting the findings of this study are available from the corresponding author (Jorge M. S. Faria) upon reasonable request.

## Supplementary Materials

The following supporting information can be downloaded at: https://www.mdpi.com/article/10.3390/plants12132438/s1, Table S1: Kovats indices for the main compounds (compounds ≥ 1%) of the essential oils (EOs) of eucalypt (*Eucalyptus globulus*), fennel (*Foeniculum vulgare*), lemongrass (*Cymbopogon citratus*), oregano (*Origanum vulgare*), peppermint (*Mentha piperita*), rosemary (*Rosmarinus officinalis*), sage (*Salvia officinalis*) and winter savory (*Satureja montana*); Table S2: Confidence intervals (95%) of the EC_50_ values determined for the essential oils (EOs), on diagonal, and EO mixtures, below diagonal.
