# Supplementary material for: First Report on the Synergistic Interaction between Essential Oils against the Pinewood Nematode Bursaphelenchus xylophilus
Source: Plants (Basel). 2023 Jun 25;12(13):2438. doi: 10.3390/plants12132438 (PMC10347196; doi:10.3390/plants12132438)
Supplement: Supplementary file 1 [file plants-12-02438-s001.zip › plants-2453906-supplementary.pdf]

Supplementary material

First report on the synergistic interaction between essential oils against the pinewood nematode *Bursaphelenchus xylophilus*

Jorge M. S. Faria[1,2*], Tomás Cavaco[1,3], Diogo Gonçalves[1,4], Pedro Barbosa[2], Dora Martins Teixeira[5,6], Cristina Moiteiro[4], Maria L. Inácio[1,7]

[1] INIAV, I.P., National Institute for Agrarian and Veterinary Research, I.P., Quinta do Marquês, 2780-159 Oeiras, Portugal; fariajms@gmail.com (J.M.S.F.), tomasfcavaco@gmail.com (T.C.), lurdes.inacio@iniav.pt (M.L.I.)
[2] MED, Mediterranean Institute for Agriculture, Environment and Development & CHANGE—Global Change and Sustainability Institute, Institute for Advanced Studies and Research, Évora University, Pólo da Mitra, Ap. 94, 7006-554 Évora, Portugal; pedronematology@gmail.com
[3] Instituto Superior de Agronomia (ISA), Universidade de Lisboa, 1349-107 Lisboa, Portugal
[4] Centro de Química Estrutural, Institute of Molecular Sciences, Departamento de Química e Bioquímica, Faculdade de Ciências, Universidade de Lisboa, Campo Grande, 1749-016 Lisboa, Portugal; diomascarenhas@gmail.com (D.G.); cmmoiteiro@fc.ul.pt (C.M.)
[5] HERCULES Laboratory, Évora University, Largo Marquês de Marialva 8, 7000-809 Évora, Portugal; dmt@uevora.pt
[6] Science and Technology School, Évora University, Rua Romão Ramalho nº 59, 7000-671 Évora, Portugal
[7] GREEN-IT Bioresources for Sustainability, Instituto de Tecnologia Química e Biológica, Universidade Nova de Lisboa (ITQB NOVA), Av. da República, 2780-157 Oeiras, Portugal
* Correspondence: fariajms@gmail.com

Table S1. Kovats indices for the main compounds (compounds ≥ 1%) of the essential oils (EOs) of eucalypt (*Eucalyptus globulus*), fennel (*Foeniculum vulgare*), lemongrass (*Cymbopogon citratus*), oregano (*Origanum vulgare*), peppermint (*Mentha piperita*), rosemary (*Rosmarinus officinalis*), sage (*Salvia officinalis*) and winter savory (*Satureja montana*).

**EO compounds**	**Kovats Index**
trans-Anethole	1283
β-Bisabolene	1509
1,8-Cineole	1036
Camphene	953
Camphor	1143
Carvacrol	1299
p-Cymene	1027
β-Caryophyllene	1428
Geranial	1270
Geraniol	1255
Isomenthone	1164
Limonene	1039
Linalool	1098
Menthofuran	1164
Menthol	1173
Menthone	1154
β-Myrcene	991
Neomenthol	1169
Neral	1240
Pinocarvone	1168
trans-Pinocarveol	1141
Pulegone	1237
α-Phellandrene	1005
α-Pinene	933
β-Phellandrene	1053
β-Pinene	981
Terpinen-4-ol	1178
Thymol	1290
α-Terpinene	1018
α-Terpineol	1189
α-Thujone	1102
β-Thujone	1110
γ-Terpinene	1062
Verbenone	1205
Viridiflorol	1590

Table S2. Confidence intervals (95%) of the EC_{50} values determined for the essential oils (EOs), on diagonal, and EO mixtures, below diagonal.

**EOs**	***Cymbopogon citratus***	***Eucalyptus globulus***	***Foeniculum vulgare***	***Mentha piperita***	***Origanum vulgare***	***Rosmarinus officinalis***	***Salvia officinalis***	***Satureja montana***
C. citratus	0.275-0.299							
E. globulus	-	-						
F. vulgare	0.105-0.239	-	0.243-0.778					
M. piperita	0.092-0.094	-	-	-				
O. vulgare	0.073-0.246	-	0.124-0.138	0.070-0.244	-			
R. officinalis	0.090-0.234	-	-	-	0.114-0.386	-		
S. officinalis	0.070-0.212	-	-	-	0.076-0.210	-	-	
S. montana	0.159-0.162	0.181-0.191	0.048-0.051	0.070-0.021	0.072-0.204	0.070-0.021	0.076-0.212	0.114-0.156
